# The Need to Develop Standard Measures of Patient Adherence for Big Data: Viewpoint

**DOI:** 10.2196/18150

**Published:** 2020-08-27

**Authors:** Przemyslaw Kardas, Isabel Aguilar-Palacio, Marta Almada, Caitriona Cahir, Elisio Costa, Anna Giardini, Sara Malo, Mireia Massot Mesquida, Enrica Menditto, Luís Midão, Carlos Luis Parra-Calderón, Enrique Pepiol Salom, Bernard Vrijens

**Affiliations:** 1 Department of Family Medicine Medical University of Lodz Lodz Poland; 2 Preventive Medicine and Public Health Department Zaragoza University Zaragoza Spain; 3 Fundación Instituto de Investigación Sanitaria de Aragón (IIS Aragón) Zaragoza Spain; 4 UCIBIO REQUIMTE, ICBAS, Porto4Ageing - Competences Center on Active and Healthy Ageing Faculty of Pharmacy University of Porto Porto Portugal; 5 Division of Population Health Sciences Royal College of Surgeons in Ireland Dublin Ireland; 6 IT Department Istituti Clinici Scientifici Maugeri IRCCS Pavia Italy; 7 Servei d’Atenció Primària Vallès Occidental Institut Català de la Salut Barcelona Spain; 8 CIRFF, Center of Pharmacoeconomics University of Naples Federico II Naples Italy; 9 Department of Pharmacy University of Naples Federico II Naples Italy; 10 Group of Research and Innovation in Biomedical Informatics, Biomedical Engineering and Health Economy, Institute of Biomedicine of Seville, IBiS / Virgen del Rocío University Hospital / CSIC / University of Seville Seville Spain; 11 International Commitee, Muy Ilustre Colegio Oficial de Farmacéuticos Valencia Spain; 12 AARDEX Group Seraing Belgium; 13 Liège University Liège Belgium

**Keywords:** patient adherence, big data, metrics, consensus

## Abstract

Despite half a century of dedicated studies, medication adherence remains far from perfect, with many patients not taking their medications as prescribed. The magnitude of this problem is rising, jeopardizing the effectiveness of evidence-based therapies. An important reason for this is the unprecedented demographic change at the beginning of the 21st century. Aging leads to multimorbidity and complex therapeutic regimens that create a fertile ground for nonadherence. As this scenario is a global problem, it needs a worldwide answer. Could this answer be provided, given the new opportunities created by the digitization of health care? Daily, health-related information is being collected in electronic health records, pharmacy dispensing databases, health insurance systems, and national health system records. These big data repositories offer a unique chance to study adherence both retrospectively and prospectively at the population level, as well as its related factors. In order to make full use of this opportunity, there is a need to develop standardized measures of adherence, which can be applied globally to big data and will inform scientific research, clinical practice, and public health. These standardized measures may also enable a better understanding of the relationship between adherence and clinical outcomes, and allow for fair benchmarking of the effectiveness and cost-effectiveness of adherence-targeting interventions. Unfortunately, despite this obvious need, such standards are still lacking. Therefore, the aim of this paper is to call for a consensus on global standards for measuring adherence with big data. More specifically, sound standards of formatting and analyzing big data are needed in order to assess, uniformly present, and compare patterns of medication adherence across studies. Wide use of these standards may improve adherence and make health care systems more effective and sustainable.

## Introduction

Despite half a century of dedicated studies, medication adherence remains far from perfect. In fact, nonadherence to medication (ie, the scenario in which patients are not taking their medications as prescribed) is still very prevalent. According to the World Health Organization (WHO), 50% of patients are estimated to deviate from their chronic treatments [1]. It has been shown to lead to poor health outcomes, increased use of health services, and increased costs (both direct and indirect, eg, due to absenteeism, lost productivity, etc), jeopardizing the effectiveness of evidence-based therapies [2]. In a recent meta-analysis, medication nonadherence was found to be associated with all-cause hospitalization (adjusted odds ratio 1.17, 95% CI 1.12-1.21) and mortality (good adherence was associated with a 21% reduction in long-term mortality risk in comparison with medication nonadherence; adjusted hazard ratio 0.79, 95% CI 0.63-0.98) in older people [3]. Thus, nonadherence is an important determinant of individual health. At the population level, it also seriously affects public health and the economy. Considering all these effects, nonadherence has been recognized by the WHO as a “problem of striking magnitude” [1].

Unfortunately, the seriousness of this problem is ever increasing. An important reason for this is the unprecedented demographic change that is taking place at the beginning of the 21st century. It affects the whole world and is particularly pronounced in Europe. According to Eurostat, currently, persons aged 65 years or older comprise 20% of the EU-28 population, and this proportion is expected to rise up to 31% by 2100. Even more striking are the statistics for very old citizens; the proportion of persons aged 80 years or older is expected to rise within the same time period from 6% (current) of the EU-28 population to 15% [4].

Longer lifespan results in an increase in the prevalence of noncommunicable chronic conditions and multimorbidity (usually defined as the coexistence of two or more chronic conditions in an individual). This, in turn, leads to the frequent use of complex therapeutic regimens and creates a fertile ground for nonadherence [5].

In order to prevent nonadherence, one needs to know the major drivers of this phenomenon. The WHO developed a model of the determinants affecting adherence and grouped them into five main sets of factors as follows: health system–related factors, therapy-related factors, condition-related factors, patient-related factors, and socioeconomic factors [1]. Based on this model of adherence, multiple nonadherence-targeting interventions have been designed and tested, but unfortunately, only few have been successful. As stated in a recent Cochrane systematic review, “current methods of improving medication adherence for chronic health problems are mostly complex and not very effective, so the full benefits of treatment cannot be realized” [6].

Indeed, medication taking is a complex behavior, and diverse determinants play different roles at the individual level. As a consequence, it seems to be unrealistic to expect that one uniform intervention will solve the problem of nonadherence in each and every case. On the other hand, there is a rising body of evidence that nonadherence could be effectively managed through the use of various innovative digital solutions [7]. Successful examples include web-based education and monitoring programs [8], clinical decision support systems using data from electronic health records (EHRs) to produce alerts [9], mobile technologies (mobile health [mHealth]), dedicated apps providing various combinations of patient monitoring, education, and facilitation of adherence [10,11], etc. Owing to digitization, for the first time in history, nonadherence may also become precisely measurable on a mass scale owing to the availability of large health care databases. This is particularly important for older populations, which is a group usually understudied in clinical trials for various reasons (eg, multimorbidity and related polymedication).

However, these promising opportunities are not fully utilized yet owing to a lack of basic widely accepted standards for measuring and managing adherence in big data. The discussion that started in 2019 at the forums of professional bodies active in the area of patient adherence research (ie, Action Group A1 “Adherence to prescription and medical plans” of the European Innovative Partnership on Active and Healthy Ageing and International Society for Medication Adherence ESPACOMP), to which the authors of this publication belong, led to the conclusion that this scenario needs to be changed. This idea corresponds very well with recent recommendations of the Heads of Medicines Agencies-European Medicines Agency joint Big Data Task Force, which called for the development of skills and the creation of capabilities to analyze big data [12]. Therefore, the aim of this publication is to establish a call for a consensus on global standards for measuring adherence with big data. More specifically, sound standards of formatting and analyzing big data are needed in order to assess, uniformly present, and compare patterns of medication adherence across studies, and thus, help scientific research, clinical practice, and public health.

## Opportunities Created for Adherence Owing to Digitization of the Health Care Sector

Digitization is a new opportunity that has, in recent times, become more frequently adopted in the health care sector. Interestingly, to date, digital solutions have been widely used outside health care, but they have only recently been employed in the field of medicine and offer great promise toward improved and more efficient care. In order to speed up this process, in 2018, the European Commission developed a plan for the digital transformation of health and care in the Digital Single Market. The plan is based on three pillars as follows: (1) securing data access and sharing; (2) connecting and sharing health data for research, faster diagnosis, and improved individualized health care services and health outcomes; and (3) strengthening citizen empowerment and individual care through digital services [13]. This plan is a part of the overarching European Strategy for Data [14]. Digitization of the health care sector creates an opportunity for using big data analytics tools and methods to assess nonadherence, improve clinical practice or health care services, and promote the use of tailored interventions. Routinely collected information on prescribing and dispensing, which are available in EHRs, pharmacy dispensing databases, health insurance claims systems, and national health systems records, enables a more thorough exploration of the relationship between adherence and health outcomes. The rising use of mHealth by patients for self-monitoring and disease management is also another potential data source for analysis. Thus, big data may represent a powerful and relatively low-cost resource for investigating important public health concerns in real-life scenarios, including the prevalence of nonadherence, its drivers, and the consequences of nonadherence. Big data can also be used to provide information for designing new interventions and targeting both prevention and management of nonadherence. Moreover, big data allow research on an incomparable scale, covering large populations (eg, primary nonadherence, a measure of unfilled prescriptions, was recently assessed in a cohort of 1.6 million Catalonian primary care patients [15] and in a national population based study in Poland [16]). Unlike medical trials, big data also provide an opportunity to assess adherence longitudinally (eg, an Estonian study analyzed a national database over a period of 15 years [17]). All this is possible without typical limitations in terms of cost, intrusiveness, and bias, which are characteristic of studies employing other sorts of data for adherence measurement and monitoring. However, at present, uniform and accepted standards of adherence measurement for big data are still lacking. Moreover, currently, big data collection is not uniformly formatted or structured for adherence measurement, which means that nontrivial operations are needed to allow for this kind of analysis. Therefore, to build a solid evidence base for adherence management across clinical settings, it is necessary to standardize adherence estimation and facilitate the appropriate use of these standards [18].

Adherence research is not the only area of research facing problems with standardization when it comes to digital health. For example, there are no global standards for EHRs [19]. Various historical, cultural, economic, and political reasons could be cited as causative factors, and despite activities of several interoperability initiatives, both public and private, this is still the case [20]. Several standardization development organizations have developed very mature and widely implemented standards, such as the Clinical Document Architecture [21] and Fast Healthcare Interoperability Resources [22] by HL7, and CEN-ISO 13606 [23], but they are limited in their interoperability. Interestingly, ISO 10781:2015 provides a reference list of functions that may be present in an EHR system, and of these, the following function tackles adherence assessment: Care Patient Support CPS 3.1 Function on “Support for Standard Assessments” [24].

A systemic review of the challenges for the use of big data in health care identified issues in data structure, security, data standardization, storage and transfer, and managerial skills, such as data governance, to be the most often provided in the current literature [25]. Practical challenges included data preprocessing and curation, model training, refinement of systems, ethical and legal issues, data privacy and security, end users understanding acceptance, etc [26,27].

Certainly, to overcome all these challenges is not easy. However, it is quite easy to illustrate why this scenario urgently needs to be changed.

## Need for Standard Big Data–Related Adherence Metrics for Research

The introduction of the Ascertaining Barriers for Compliance taxonomy (ABC taxonomy) and new adherence terminology (named after the dedicated European research project “*Ascertaining Barriers for Adherence*”) made the first big step forward in terms of standardization by defining three essential components of adherence. These components are as follows: (1) initiation (taking the first dose of the prescribed medication); (2) implementation (taking medication as prescribed); and (3) discontinuation (stopping treatment) [28]. Following this, the recently introduced EMERGE (*ESPACOMP Medication Adherence Reporting Guideline* developed under the umbrella of the International Society for Medication Adherence, COMpliance, and Persistence [ESPACOMP]) provides guidance, along with a checklist for reporting results of studies on medication adherence [29]. Other interesting activities in this area include the work initiated with the support of the Government of Spain and the European project StandICT.eu to generate an extension of the SNOMED CT terminology for the domain of adherence [30] and the proposal to include terms of adherence in the amendment to ISO 13940 (system of concepts to support continuity of care) currently in progress in ISO TC 215 [31].

However, there are still many challenges with the use of big data for adherence assessment. Without standard metrics, the same data may lead to diverse results, as clearly depicted in the study by Malo et al, which found different mean adherence values and proportions of adherent patients when using medication possession ratio versus proportion of days covered [32].

To date, numerous studies on adherence have been undertaken, using diverse approaches to data analysis, which have led to mixed results. Most often, pharmacy records are used to measure adherence in terms of implementation and discontinuation [33]. EHR data have also been used for effective prediction of medication adherence trajectories [34], which has evoked certain discussions [35]. Menditto et al managed to integrate and analyze six databases from three countries, which allowed for a fair comparison of medication adherence across the various countries [36]. Another study managed to assess and compare adherence to chronic medication across three European cohorts of older people by developing a common protocol and using structured documents for sharing and applying methodologies [37].

Some attempts to introduce standards to adherence assessment in big data have been made by the International Society for Pharmacoeconomics and Outcomes Research (ISPOR). The ISPOR Medication Adherence and Persistence Special Interest Group produced recommendations for assessment of initial medication adherence [38] and proposed a checklist for medication adherence studies using retrospective databases [39]. Arnet et al [40] and Raebel et al [41] proposed standard definitions and their operationalization to quantify adherence to medication from electronic databases. Lehnman et al [42] and Williams et al [43] provided some basic guidance regarding the use of pharmacy refill data to assess adherence. At the same time, a systematic review of publications on adherence in older Americans identified as many as 20 differently named measures of adherence derived from pharmacy claims data [44]. Even more interestingly, some adherence measures derived from big data are already in use for incentivizing health care providers to consider long-term health outcomes. In the United States, the Centers for Medicare & Medicaid Services adopted several quality measures using the threshold of the proportion of days covered of ≥0.8 for the drugs under measurement, for a period of 12 consecutive months [45]. In fact, there is evidence that this improves adherence [46].

Moreover, another important question that needs to be addressed in future standardized measures of adherence in big data is “what is the subject of adherence assessment: a drug, a condition, or a patient?” In other words, how to measure adherence to multiple medications prescribed for the same condition and/or to various conditions in patients with multimorbidity [47]. Indicators designed and widely used to evaluate single-medication adherence are not necessarily valid for the assessment of adherence to polypharmacy regimens [48]. For example, a study assessing adherence in individuals belonging to the Epichron cohort returned highly diverse results for various drug classes as follows: 72.4% for antidiabetics versus only 44.3% for lipid-lowering drugs [49]. A recent systematic review found serious inconsistency in the measures used to estimate adherence and persistence to multiple cardiometabolic medications [50], while another review concluded that “there appears to be no standardized method to measure multiple medication adherence” [51]. For sure, further research is needed in this respect and is particularly important given the aging population.

In summary, the major disadvantage of the current lack of widely accepted standards for adherence assessment is the difficulty in comparing and interpreting scientific studies' results. Uniform adherence measurement and a common ontology urgently need to be developed in order to support research and enable real-life implementation of study findings [40,52,53]. This is also necessary for cross-study comparisons and fair benchmarking of adherence-targeting interventions.

## Need for Standard Big Data–Related Adherence Metrics for Clinical Practice, Public Health, and Health Policy

Big data and the development of a standardized measure of adherence may enable more reliable and valid investigations into the association between nonadherence and health outcomes. To date, there is no consensual standard for what constitutes adequate adherence. In practice, 80% is often used as a cutoff to classify “good adherence,” but scientific evidence for this threshold is unclear. In fact, a systematic review investigated medication adherence thresholds in relation to clinical outcomes and found the included studies to be highly heterogeneous, and it could not confirm or reject the validity of the historical 80% cutoff threshold for adherence [54]. Moreover, many treatments are also preventative, and it may take a very long time to determine any therapeutic benefit at all from such treatments.

Various interventions have been designed to prevent and manage nonadherence in real-life settings. Unfortunately, despite objective need, these interventions are generally underused. Lack of standardized comparable measures of adherence is one of the major barriers to the objective selection of the most effective and cost-effective interventions [2] and the scaling-up of the best practice. Only with reliable and valid measures can nonadherence be tracked along a timeline, allowing the assessment of the long-term effects of particular interventions and the benchmarking of their effectiveness. Standard measures and guidelines to assess adherence could also facilitate the introduction as well as the assessment of the effectiveness of incentives to promote adherence at the patient, provider, and payer levels and the ability to target individual risk factors at the various health care provider levels [55].

Standardized adherence measures employed in big data sets may also provide insights into the reasons why patients do not adhere to their prescribed medication regimens. This is of utmost importance as a review of systematic reviews identified 771 individual factor items as possible determinants of nonadherence, concluding that “lack of standardized adherence definitions and use of poor measurement methods resulted in many inconsistencies in the findings and many of the identified factors had an inconsistent effect on adherence” [56].

Thus, big data sets are useful to assess adherence in different profiles of drug users, analyze the factors related to adherence, explore causes of discontinuation, and compare results across different populations. With this information, drug users at the highest risk of nonadherence can be identified, and tailored interventions can be designed and implemented. It is of paramount importance to consider that most of the current interventions to address adherence are using or are based on information technology (IT) solutions. These, however, are not currently based on standardized measures of adherence [55]. Another potential technology approach for prediction and assessment of adherence is artificial intelligence (AI). With AI, big data may be analyzed both retrospectively and in real time, allowing for more personalized health care. However, a major hindrance to the adoption of AI is, yet again, the lack of sound operational measures of adherence to properly train the algorithms.

Along with individual health, public health is sure to benefit from the introduction of standardized measures of adherence. Such measures will enable comparison of adherence rates within and across different countries, populations, and disease groups, allowing for fair benchmarking of interventions, better planning, and practical implementation. In fact, more often, medication adherence is accepted as a measure of the quality of care provided by physicians, as well as the quality and effectiveness of the entire health care system [55,57]. Moreover, in this area, a lack of standardized measures causes the problem of nonadherence to be often overlooked in national agendas. Currently, only few countries systematically monitor adherence. Therefore, the recent Organization for Economic Co-operation and Development (OECD) report calls for standardization in order to allow for international benchmarking [55].

The recent outbreak of the COVID-19 pandemic has shown the extraordinary role that infoepidemiology (ie, information epidemiology) can play in the management of major public health problems [58]. Let this lesson be an inspiration for the wider adoption of digitization in health care in general and the faster utilization of the potential of big data for the management of adherence in particular.

## Conclusions

Ongoing digitization of the health care sector and availability of big data repositories create an unprecedented opportunity to study patient adherence on a mass scale both retrospectively and in real time. Obvious benefits that could be derived for science, as well as individual and public health, are hindered by the current lack of standards for adherence-related data analysis. What sort of standards need to be agreed upon to change this scenario? First, there needs to be a standard format for the data collected in big data databases, such as EHRs and prescribing and dispensing registers, to allow for smooth and effective assessment of adherence parameters. Second, sound metrics need to be developed to process the raw data. Third, the standards of presentation of adherence measures being assessed within big data need to be agreed upon.

Bearing in mind the troublesome history of health care sector digitization, this plan may appear to be ambitious. However, the authors of this paper are motivated to face the challenge and develop these highly needed global standards, discuss them with the scientific world, and finally, agree on a common consensus. Therefore, interested individuals are invited to join our efforts within the new initiative that we want to call DIGI.PASs (Patient Adherence Standard measures to be used with big data collections available in DIGItal repositories).

## Abbreviations

AI: artificial intelligence
EHR: electronic health record
EMERGE: ESPACOMP Medication Adherence Reporting Guideline
ESPACOMP: European Society for Patient Adherence, COMpliance, and Persistence
ISO: International Organization for Standardization
ISPOR: International Society for Pharmacoeconomics and Outcomes Research
IT: information technology
WHO: World Health Organization
